# Longitudinal Effects of Transcranial Direct Current Stimulation on Daily Rejection-Related Emotions in Borderline Personality Disorder: An Ecological Momentary Assessment Study Protocol

**DOI:** 10.3390/brainsci15050530

**Published:** 2025-05-20

**Authors:** Chiara De Panfilis, Alessandro Lisco, Kevin B. Meehan, Maria Lidia Gerra, Emanuele Preti, Paolo Riva, Leonor Josefina Romero Lauro

**Affiliations:** 1 Unit of Neuroscience, Department of Medicine and Surgery, University of Parma, 43126 Parma, Italy; 2 Ospedale Maria Luigia, 43126 Monticelli Terme, Italy; lisco@ospedalemarialuigia.it; 3 Department of Psychology, Long Island University, Brooklyn, NY 11201, USA; kbmeehan@gmail.com; 4 Department of Mental Health, Parma Local Health Agency, 43126 Parma, Italy; magerra@ausl.pr.it; 5 Department of Psychology, University of Milan-Bicocca, 20126 Milan, Italy; emanuele.preti@unimib.it (E.P.); paolo.riva1@unimib.it (P.R.); leonor.romero1@unimib.it (L.J.R.L.); 6 Bicocca Center for Applied Psychology (BICAPP), University of Milan-Bicocca, 20126 Milan, Italy; 7 NeuroMi—Milan Center for Neuroscience, University of Milano-Bicocca, 20126 Milan, Italy

**Keywords:** borderline personality disorder, transcranial direct current stimulation, rejection-related emotions, right ventrolateral prefrontal cortex, ecological momentary assessment

## Abstract

Background: Borderline Personality Disorder (BPD) is a debilitating mental health condition characterized by emotional dysregulation and interpersonal dysfunction, with perceived social rejection exacerbating these issues. Emerging evidence suggests that a single session of transcranial direct current stimulation (tDCS) over the right ventrolateral prefrontal cortex (rVLPFC) may decrease the unique tendency of BPD patients to feel rejected even when socially included during a laboratory task. Objectives: This protocol outlines a double-blind, sham-controlled study evaluating the longitudinal effects of repeated anodal tDCS over the right ventrolateral prefrontal cortex (rVLPFC) on rejection-related emotions (RRE) during real-life social interactions in individuals with BPD. Methods: Sixty BPD patients will be randomized to receive real or sham tDCS across 10 daily sessions, coupled with an ecological momentary assessment (EMA) protocol capturing emotional and behavioral responses to real-life social interactions over four timepoints: baseline, during treatment, ten days post-treatment, and three months post-treatment. Primary outcomes include changes in RRE, with exploratory analyses examining feelings of social connection, aggressive tendencies, trust toward others, and interpersonal and affective dynamics. Multilevel modeling will assess temporal and group-level effects. Expected Results and Impact: This study aims to establish the efficacy of tDCS in reducing BPD patients’ negative emotional response in real-life social situations and to determine whether such effects are maintained in time. The findings could advance the clinical application of tDCS as an adjunctive intervention to alleviate social–emotional impairments in BPD, addressing gaps in current treatment approaches and guiding future research into the neural mechanisms of social emotion regulation.

## 1. Introduction

Borderline Personality Disorder (BPD) is a serious, prevalent mental disorder with an adolescent onset. It is linked to high functional impairment, extensive treatment utilization, high mortality rates by suicide and other causes, and high societal costs [1]. Disturbed and instable interpersonal relationships are a core and debilitating feature of BPD [2,3]. Difficulties in systems for social processing are considered a key determinant of BPD interpersonal dysfunction [4]. Specifically, BPD patients exhibit a unique, biased view of others as rejecting them even when they are socially included by others, reporting greater negative affect following social inclusion than non-BPD groups [5]. This altered perception of social inclusion might contribute to the marked instability in close relationships in BPD, characterized by an unrealistic fear of abandonment and shifts between devaluation and idealization of others [1]. Studies using Cyberball [6], a virtual ball-tossing game simulating different levels of social inclusion, demonstrated that BPD patients, as compared to healthy controls, reported being more threatened in their need to belong and having greater rejection-related emotions not only when objectively excluded, but also when fairly included [7,8,9,10,11]. Only a laboratory condition of over-inclusion, in which participants were more than fairly included by others, was associated with a reduction in negative emotions to levels comparable to those of control participants, but not with similar degrees of social connection nor satisfaction with fundamental needs [10,12,13]. Importantly, this response pattern is not explained by an altered cognitive, objective rejection perception during the varying inclusionary conditions, which is intact in BPD [14]. Rather, BPD patients seem to exhibit a deviant emotional reactivity and affective evaluation in social scenarios, which makes them feel rejected and socially disconnected from others even during “fair”, adequate social exchanges [13,14].

In humans, actual social exclusion elicits painful feelings and activates brain regions involved in emotion regulation. Evidence suggests that the right ventrolateral prefrontal cortex (rVLPFC) may play a key role in the regulation of the negative emotional responses to social exclusion simulated by the Cyberball experiment [15,16,17,18]. Among healthy subjects, stimulating the rVLPFC using non-invasive neurostimulation techniques, such as anodal transcranial direct current stimulation (tDCS), decreases social pain and aggressive reactions following the Cyberball social exclusion condition [19,20]. Conversely, the downregulation of the same area using cathodal tDCS increases the negative emotions experienced following social exclusion [21].

Building on this evidence among non-clinical samples, we recently evaluated [14] whether, in BPD patients, a single session of anodal tDCS over the rVLPFC could reduce rejection-related emotions (RREs) following those Cyberball conditions associated with perceived social exclusion (which for those with BPD, but not healthy controls, also included fair play conditions). In a double-blind, sham-controlled, randomized pilot trial including forty patients with BPD, we found that, as compared to a sham stimulation, real anodal tDCS over rVLPFC reduced RREs during both social exclusion and fair inclusion, but not during over-inclusion. Specifically, real tDCS was effective in decreasing the levels of RREs experienced after fair inclusion to levels comparable to those experienced in the over-inclusion condition, while BPD patients receiving the sham stimulation still reported greater RREs in the inclusion condition than in the over-inclusion condition. These laboratory results suggest that, in BPD, anodal tDCS over the rVLPFC may be effective in decreasing BPD patients’ unique tendency to feel rejected even in fairly including interpersonal contexts, which is a key determinant of BPD patients’ interpersonal and psychosocial difficulties.

Overall, these findings may pave the way for further investigations on the application of tDCS over the rVLPFC as a treatment strategy in BPD. While effective psychotherapies for BPD decrease symptom severity at a clinically important level, improvements in social functioning are small and do not meet the criterion of minimal clinically relevant change [22]. In addition, medications have only modest and inconsistent effects in BPD and do not alter the course of the disorder [1]. In this context, non-invasive brain stimulation techniques may prove useful as an adjunctive to psychotherapy. Specifically, tDCS is a safe, inexpensive, well-tolerated, and easy to use device [23,24].

Previous tDCS research in BPD targeted mainly the dorsolateral PFC, showing a significant reduction in impulsivity and emotional dysregulation, although the variability in experimental procedures and outcome measures makes direct comparisons with the few existing studies difficult [25,26]. Our study [14] showed the effectiveness of anodal tDCS over rVPFC in downregulating feelings of rejection, pointing toward the rVLPFC as an alternative target to tap emotional reactivity. To our knowledge, however, no study has yet investigated whether these results, achieved during a laboratory experiment simulating varying degrees of social inclusion, might also extend to the achievement of more balanced real-life interpersonal exchanges (e.g., via a far-transfer effect).

Therefore, the present study evaluates whether repeated sessions of anodal tDCS over the rVLPFC result in long-lasting improvements in the regulation of RREs that generalize to daily interpersonal interactions for those with BPD. For this aim, BPD patients will be randomized to receive either a real or sham tDCS protocol of repeated daily stimulation for 10 working days. They will be asked to assess their daily emotional responses to real-life significant social interactions using an experience sampling of interpersonal interactions, or ecological momentary assessment (EMA) at multiple timepoints (e.g., before, during, and both 10 days and 3 months after the end of the tDCS protocol).

Based on previous evidence that among HC anodal tDCS over the rVLPFC decreases aggressive reactions following social exclusion [20], and that BPD patients feel disconnected by others even in including and over-including scenarios [10] and exhibit extensive impairments in trust processes [27], the EMA protocol will also assess participants’ momentary feelings of social connection and trust toward others, their level of aggressive tendencies, and their perception of their own and their interaction partner’s behavior.

In our prior work, we showed the utility of EMA to evaluate affective and interpersonal changes in patients with BPD treated in psychotherapy, suggesting not only that the occurrence and volatility of daily negative affects decreased over time periods but also that this was accompanied by shifts in interpersonal perceptions [28]. BPD patients (N = 45) made simple ratings of their perceptions of interpersonal warmth/dominance (i.e., is the behavior friendly or unfriendly, agentic or passive?) and affect valence/arousal (i.e., is the emotion happy or sad, intense or quiet?) in both themself and others; multilevel models examined shifts in covariations between these dimensions over the time period during an intervention. Related to the present study, over the course of treatment, BPD patients’ momentary perceptions of others as being more affectively aroused and interpersonally dominant was increasingly connected to seeing them as more friendly. Put differently, the tendency to view an interpersonal event as warm and friendly was associated with a wider range of other’s behaviors and affects, now including even those perceived to be more emotionally intense and agentic. Thus, EMA provided a powerful tool for demonstrating whether experiences of warmth/closeness were associated with highly contextual versus broad interpersonal experiences, as well as shifts in those associations over time via intervention.

Our main hypothesis is that participants receiving the actual tDCS protocol will report decreased RREs over time periods during daily interpersonal interactions (significant change T0–T1, sustained in T2) than participants receiving the sham protocol. We will also explore whether this decrease is also sustained 3 months after the end of the tDCS protocol (T3).

Furthermore, as a further exploratory aim, we will assess BPD patients’ feelings of social connection, aggressive tendencies, trust feelings, and interpersonal and affective dynamics during daily interpersonal interactions. For these exploratory aims we hypothesize that participants in the real tDCS group, as compared to participants in the sham group, will report greater feelings of social connection, lower aggressive tendencies, and higher trust toward others, and will rate their perceptions of their daily interpersonal events as increasingly warm/friendly and inclusive over time (significant change T0–T1, sustained T2–T3). We also hypothesize shifts in covariations among interpersonal/affective dimensions over time (significant change T0–T1, sustained T2–T3) such that participants perceive warmth/friendliness across a wider range of daily experiences of inclusion, affective intensity, and interpersonal dominance in the real tDCS group vs. sham.

As a last exploratory aim, we will assess the tDCS effects on BPD symptoms, reflective functioning and emotion regulation over time: specifically, we hypothesize that real tDCS will lead to an improvement in these dispositional dimensions (significant change T0–T2, sustained T2–T3).

## 2. Materials and Methods

### 2.1. Sample Recruitment

**Figure 1:**
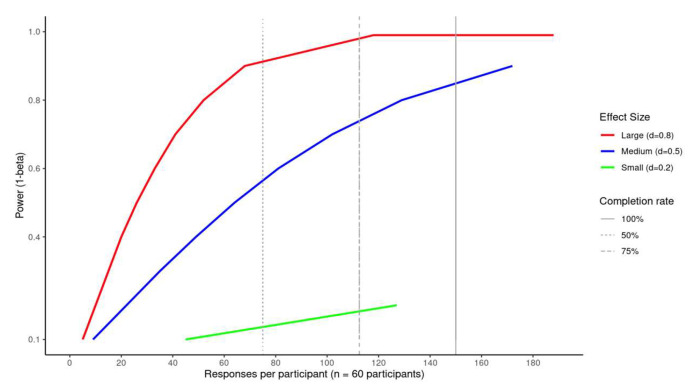
Power curve for multilevel model. Note: Power analysis for N = 60, 30 days, 5 responses, ICC = 0.40 [34].

This study will involve 60 BPD patients who will be recruited among outpatients seeking treatment at an Italian community-based Department of Mental Health, after obtaining approval from the Local Ethical Authority. An a priori power analysis, performed with G-Power 3.1 [29], considering a F-ANOVA mixed repeated measures, with a between-subjects factor (tDCS group, 2 levels: sham and real tDCS) and a within-subjects factor (time, 3 levels: T0 = baseline, T1 = during the tDCS protocol, T2 = 10 days after the end of the tDCS protocol; a subsample collected T3 = 3 months after the end of the tDCS protocol were not included in the power analysis), resulted in a sample size of 30 participants (15 per group) to detect reliable outcome measures with α = 0.05, effect size (d) = 0.28, and power = 0.95. Notably, the effect size of 0.28 was based on the effect sizes of previous works from the research group [19,20,21]. We doubled the sample size suggested by the power analysis to account for the number of participants needed in the EMA protocol. While our EMA protocol was more intensive than average, a meta-analysis of response compliance in EMA studies with BPD patients showed that they were tolerant of more intensive prompt schedules and evaluation days, with a 79% compliance rate [29]. Further, we have adopted many design features associated with greater compliance in EMA studies of psychiatric patients, including regular prompt intervals spaced out over a longer period of time [30]. There are power increases as the number of observations increases in a repeated measures design; in the 30-day EMA protocol (T0-T2), participants rated approximately 150 events (5 events/day × 10-day bursts × 3 bursts) (Figure 1). We assumed an average compliance rate of 80% for all EMA prompts (120 observations per participant for T0–T2), thus accounting for missing data. Figure 1 provides a power curve for multilevel models, showing that 60 participants provided adequate power (>0.75) to detect a medium (d > 0.5) effect, assuming an 80% completion rate during T0-T2 with an ICC value of 40%, as per our prior studies [28,31,32,33]. The subsample of participants who agreed to complete a 3-month follow up (we expected 50% of the original sample), assuming a compliance rate of 80% for all EMA prompts, resulted in an additional 40 observations per subsample participant at T3.

All participants were first evaluated by a trained investigator (senior-level resident in psychiatry) during an individual diagnostic screening to determine their eligibility for the study. Further, information on socio-demographic features, personality features (see Trait measures below), and ongoing pharmacological treatments was collected. Only patients meeting the inclusion criteria and not having any exclusion criteria for study participation were enrolled in the study. The inclusion criteria for both groups was a BPD diagnosis according to DSM-5 [35] criteria, as established by the Structured Clinical Interview for DSM-5 PD (SCID-5-PD) [36], and age 18–65. The exclusion criteria were as follows: a diagnosis of psychosis, active substance dependence, or an active mood disorder, as established by the Structured Clinical Interview for DSM-5, Clinician version (SCID-5-CV) [37]; cognitive impairment (based on clinical judgment); tDCS exclusion criteria, such as unstable medical conditions or the diagnosis of neurologic or heart diseases; presence of metallic implants, pacemakers, or acoustic prostheses; and some specific drug treatments, mainly tricyclic antidepressant therapies [25]. After the screening and the baseline assessment, all participants were then trained on the use of the app for the EMA and on the use of the experimental measures implemented in the protocol (see Section 2.3. *EMA protocol*).

### 2.2. Trait Measures

Patients will be individually administered the following questionnaires to evaluate their symptoms and their dispositional ability to deal with rejection issues and emotion regulation:

Difficulties in Emotion Regulation Scale (DERS) [38], a 36-item self-report questionnaire (Likert scale, 1–5) designed to assess multiple aspects of emotional dysregulation. DERS subscales investigate the non-acceptance of emotional responses, difficulties engaging in goal-directed behavior, impulse control difficulties, lack of emotional awareness, limited access to emotion regulation strategies, and a lack of emotional clarity.

Adult Rejection Sensitivity Questionnaire (A-RSQ) [39], a self-report questionnaire that presents respondents with 9 potential rejection situations. For each scenario, participants indicate on a 6-point Likert scale their level of expectation of rejection and of anxiety about rejection. For the current study, we added a third question about participants’ anger about rejection [40,41], leading to three different subscales: expectations of rejection, anxiety about rejection, and anger about rejection [42].

Reflective Functioning Questionnaire (RFQ) [43,44], a self-report, 8-item questionnaire (Likert scale, 1–7) assessing reflective functioning. The RFQ has two subscales, each containing 6 items, assessing certainty and uncertainty about the mental state of oneself and others.

Borderline subscale of the Personality Assessment Inventory (PAI-BOR) [45], a 24-item, widely known, and reliable measure (Likert scale, 1–4) for borderline features, such as affective instability, identity problems, negative relationships, and self-harm.

All these questionnaires are well-validated measures that are extensively employed in BPD populations to evaluate their specific difficulties in emotion regulation, rejection issues, reflective functioning, and symptoms.

Patients will complete again the above-mentioned questionnaires at T2 and at T3, to explore whether anodal tDCS over the rVLPFC might lead to potential changes in these dispositional measures as well.

### 2.3. EMA Protocol

**Figure 2:**
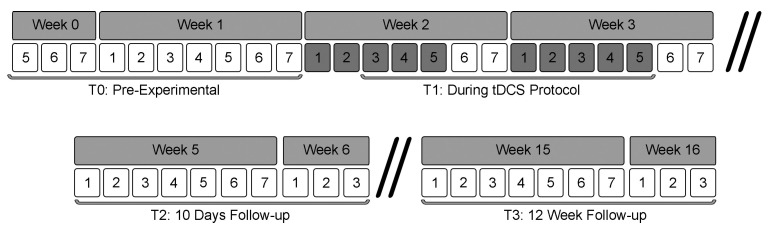
Experimental phase flowchart. Brackets indicates EMA time period, while dark gray numbered boxes indicate the weekday where participants will receive either the real or sham tDCS.

To measure the emotional and behavioral response stemming from participants’ everyday social relations, participants, after being trained on the use of an app on their smartphones, will undergo an EMA protocol in four different assessment periods, each lasting 10 days (Figure 2). The specific details are as follows:

T0: starting 10 days before the tDCS protocol to assess the baseline behavioral and emotional responses to social relations;

T1: during the 10 days of the tDCS protocol, in order to assess the effects of tDCS during the protocol;

T2: 10 days after the end of the tDCS protocol, to assess if the potential effects persist even after the end of the tDCS protocol.

T3: 12 weeks following the end of the tDCS protocol for 10 days, to assess if the potential effects persist in the long-term after the end of the tDCS protocol.

For each day of the EMA protocol, participants will receive five prompts from an app on their smartphones at five different times of the day: two in the morning (around 9 to 12.30 pm), two in the afternoon (around 15 to 19 pm), and lastly, one in the evening (around 20 to 22 pm), asking them to assess the last significant social encounter of the day (lasting at least 5 min). Moreover, participants will be asked to spontaneously report their reactions immediately after any significant social encounter (lasting at least 5 min) at least three times/day. The average EMA survey completion time in our prior study [28] was 2 min 43 s, or about 15 min per day.

For both signal-initiated and event-initiated EMAs, participants will be directed to an online survey that will ask respondents to fill in the following measures:

Primary Outcome Measure: Rejection-related emotions. Assessed by the Rejected Emotions Scale [46]. Participants will rate how they felt during *Cyberball* on a number of adjectives measuring six emotions: anger, happiness, hurt, rejection feelings, anxiety, and sadness. These ratings will be made on a 7-point scale ranging from “not at all” to “extremely”. For the sake of compliance and brevity, the original scale, assessing the different dimensions with 4 items each, has been shortened to a single item assessing the singular dimensions, for a total of 6 items.

Exploratory outcome measures:

Interpersonal and Affect Assessments.

Patients will make event-contingent recordings following any interpersonal interaction lasting 5 min or longer. Ratings include their own interpersonal behavior (self-agency, self-communion) as well as their perception of their interaction partner’s interpersonal behavior (other-agency, other-communion). Higher scores indicate greater perceived dominance (from unassured–submissive to assured–dominant) and communion (from cold–quarrelsome to warm–agreeable). Patients will also rate their own affect (self-activation, self-valence) as well as their perception of their interaction partner’s affect (other-activation, other-valence). Higher scores indicate higher perceived activation (from quiet–passive to active–energized) and higher perceived valence (from sad–upset to happy–pleased).

Social connection. Assessed by the Inclusion of Other in the Self scale (IOS) [47], a single-item, pictorial measure of the psychological overlap between the self and the other. Higher scores indicate a higher degree of social connection. Participants will rate their impression of closeness with the other person during the interpersonal event by choosing among 7 images. Each image shows two diagrams, the former related to “yourself”, the latter related to “the other”, which can be completely separated (“A”) up to partially overlapped (“G”).

Trust will be assessed by asking the participants “In this social interaction, how much did you trust the other person?”. This measure will be scored on a 7-point scale ranging from “not at all” to “extremely”.

Aggressive temptations. Assessed by the Aggressive Temptation Scale—short version (ATS) [48], a 6-item questionnaire (1–7 Likert scale) measuring participants’ self-reported temptation to engage in aggressive behaviors (e.g., humiliating another person or ignoring another person). Particularly, participants will be reminded that this scale will not ask whether they would have performed each behavior, but rather the degree to which they would have been tempted to do each one during the interpersonal event they are rating.

### 2.4. Transcranial Direct Current Stimulation (tDCS) Protocol

Participants will be randomly assigned to two stimulation conditions: anodal (real) or sham tDCS over the rVLPFC. The tDCS treatment, either real or sham, will last 10 working days and comprise 20 min daily sessions. The stimulation will be applied at an intensity of 1.5 mA, with electrodes of 25 cm^2^ (anode, current density of 0.06 mA/cm^2^) and 35 cm^2^ (cathode, current density 0.042 mA/cm^2^) in line with the safety standards of tDCS [24]. We opt for differently sized electrodes to increase the focality of the stimulation [24]. In the sham modality, all the stimulation parameters (electrode sizes and montage, current intensity, number of sessions) will be kept identical, except for the stimulation duration, which will last only 20 s, with 30 s of rump-in/out as in the real condition to maintain the same physical sensations, thus keeping the participants blind to the tDCS condition [24,49]. An intracephalic montage will be adopted, with the anode placed over the rVLPFC, while the cathode will be placed on the contralateral supraorbital region.

**Figure 3:**
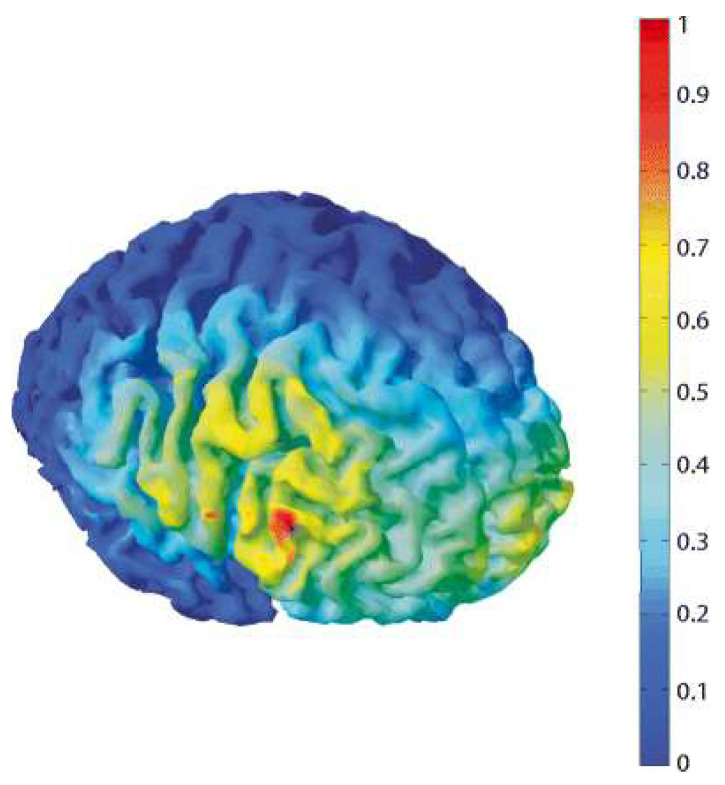
Computational model of a tDCS-induced electric field. A simulation of the electrical field induced by tDCS over the rVLPFC was computed using Comets. The anode (25 cm^2^) was placed over the rVLPFC, corresponding to the F6 electrode according to the 10–20 EEG system. The cathode (35 cm^2^) was placed on the contralateral supraorbital area. Red parts indicate the strongest electrical field, occurring over the lateral and ventral portion of the right prefrontal cortex.

We opt for an intracephalic montage based on prior research on tDCS clinical use [50,51], and to maintain consistency with the montage and tDCS parameters that effectively reduced pain-related emotions in our previous work [19,20,52]. The same electrode positioning procedure, based in the EEG 10-20 system of our previous studies [19,20,52], will be employed. Specifically, the anode will be positioned over F6 (MNI coordinates: 58, 30, 8), which corresponds to the rVLPFC. The effectiveness of such a montage to target the rVLPFC has been tested employing the free software COMETS v.1.04 (http://www.COMETStool.com, accessed on 15 March 2025) [53] to run a computational model of the current flow (figure below taken from Riva et al., 2017 [52]). This model showed that the strongest electric field was concentrated around the cortical area beneath the target electrode (Figure 3).

The tDCS device (DC-STIMULATOR, NeuroConn GmbH, Ilmenau, Germany) includes a study mode for a double-blind procedure. Namely, a numeric code (four digits), corresponding to either anodal or sham tDCS, will start the stimulation, thus preventing the awareness of the stimulation condition in both participants and the research assistant—neither of whom is aware of the correspondence between the code and the type of stimulation that will be delivered. More specifically, the research assistant will read on participants’ log files one of two codes for each new participant. Unbeknownst to the research assistant who delivers the tDCS, two codes will trigger anodal tDCS, the other two will trigger sham stimulation. Thus, the proposed study will be a double-blind study, meaning that both patients and investigators involved in the tDCS sessions will be blinded to the stimulation conditions (real or sham tDCS). Before starting the experiment, an investigator different from the one who will apply the tDCS stimulation will set the tDCS parameters for real and sham stimulation by using two different numerical codes. Such codes will be saved in an unmodified mode in the tDCS device so that at the beginning of each session, according to the randomization, active or sham stimulation will be chosen by only considering their corresponding codes. The unmodified mode rules out the possibility that codes and stimulation parameters will be changed during the experiment. To further check for participants blindness, at the end of the treatment, a questionnaire inquiring about the participant’s guess on the real vs. sham condition assignment and eventual side effects of the stimulation will be administered.

tDCS will be delivered during the *Cyberball* paradigm, a virtual ball-tossing game that reproduces different levels of social inclusion by manipulating the percentage of tosses that participants receive from two other computer-controlled co-players. After about five minutes of stimulation, participants will play first the social inclusion (33% of throws received), then the social exclusion (10% of throws), then the over-inclusion (45% of throws) conditions of the game. The order of these experimental phases is fixed, ensuring that the game ends with the over-inclusion condition, which is typically not associated with negative emotions in BPD [10,13], for ethical reasons. The choice in the study design to deliver tDCS during the *Cyberball* stimulation design is based on recent evidence on the state-dependency of tDCS, pointing to the opportunity to timelock the stimulation to a concurrent activity involving the stimulated target area in order to achieve specific effects [54,55,56]. tDCS experiments will be conducted by trained investigators and participants can stop the stimulation at any time if they want to. TDCS is not invasive, with few adverse effects, mostly consisting of an itching sensation during the ramp-in phase of the current [57]. At the end of the tDCS treatment, participants will complete a questionnaire to investigate the eventual side effects and to control for tDCS blinding effectiveness [58]. Checking blindness effectiveness indeed becomes more crucial when multiple sessions are included in a protocol. In particular, the questionnaire [58] explores participants’ beliefs on tDCS condition assignment. The frequency of correct vs. incorrect guesses will then be compared between the real vs. sham control group by means of a Chi-square test.

All subjects will be extensively debriefed upon completion of the study. They will receive detailed information about the tDCS protocol and its purpose and will have the opportunity to have their data deleted should they so wish.

### 2.5. Statistical Considerations and Data Analysis

Given the nested nature of the variable explored in the present study, the primary aim of the study will be examined by means of a 3-level multilevel model as follows: Level-1 for within-subject variability, Level-2 for the different assessment times (T0, T1, T2, T3), and Level-3 (between-group variability) for the tDCS experimental condition (real anodal tDCS vs. sham tDCS). This approach allows us to account for within-person variability across time, and between-subjects and between-conditions variability.

## 3. Expected Results and Discussion

The primary objective of this study is to evaluate whether 10 sessions of anodal tDCS over the rVLPFC decreases BPD patients’ unique tendency to emotionally react as if being rejected during most social encounters in daily life. A secondary, exploratory objective is to evaluate whether the tDCS protocol further increases BPD patients’ perception of others as warm/friendly, enhances their feelings of social connection and trust toward others, and decreases their aggressive tendencies in daily interpersonal interactions.

This will be accomplished by performing a double-blind, sham-controlled study to evaluate whether repeated sessions of tDCS over the rVLPFC ameliorate BPD patients’ emotional and interpersonal responses to their social encounters. The EMA protocol, with both event-initiated and signal-initiated assessments, will ensure the longitudinal experience sampling of real-life interpersonal events. Participants’ RREs and affective and behavioral responses following daily significant interpersonal exchanges will be evaluated before, during, and after the brain stimulation sessions. Building from previous evidence that a single session of tDCS over the rVLPFC reduces BPD patients’ bias to affectively interpret fair social inclusion as rejection in a laboratory setting [14], we aim to determine whether repeated sessions of active tDCS over the rVLPFC would decrease this biased affective interpretation of any social interaction as rejection even in daily life.

We expect that BPD patients undergoing the real tDCS protocol will show lower levels of RREs elicited by daily social interactions compared to sham controls and that this reduction in RRE will be sustained even 10 days after and up to 3 months after the end of the tDCS protocol. Consistently, we also expect that the tDCS protocol will improve BPD patients’ perception of their interactions as being increasingly warm, friendly, and inclusive over time periods across a wider range of daily experiences of inclusion, affective intensity, and interpersonal dominance, thus broadening their perception of interpersonal behaviors and affects. Specifically, participants in the real tDCS group are expected to rate their own and their interaction partners’ behaviors as being increasingly warm and affiliative, with a greater perception of friendliness and inclusion compared to the sham group. We also anticipate that affective responses during daily social interactions will shift over time, with participants in the real tDCS group reporting lower negative affect and higher positive affect during social encounters compared to the baseline and the sham condition.

Regarding trust, our hypothesis is that repeated tDCS, by reducing negative emotional responses to social interactions, could favor an increased sense of trust toward others, reflected in higher trust ratings during interpersonal interactions over time. Since BPD patients often perceive social interactions as unpredictable or threatening, we expect that tDCS will mitigate this tendency, leading to a more stable and positive perception of others’ intentions and behaviors.

Therefore, we expect that repeated sessions of tDCS over the rVLPFC will lead to enduring improvements in emotional and interpersonal regulation in daily life in BPD. Specifically, this will be reflected in lower rejection-related emotions, enhanced social connection, increased trust, and more positive affective and interpersonal experiences across a broad range of social encounters.

Importantly, previous laboratory studies using the *Cyberball* paradigm demonstrated that BPD patients feel socially disconnected by others and more threatened in their need to belong, even in a condition of extreme inclusion [10,12,13], and this response pattern is not affected by a single session of anodal tDCS over the rVLPFC [14], suggesting that this subjective experience of insufficient social connection irrespective of actual social acceptance might be an enduring, core feature of BPD, which is unlikely to be affected by a single session of rVLPFC stimulation. Consistently, loneliness, defined as the discrepancy between desired and experienced social connectedness, is regarded as a central feature of BPD and a key driver of BPD patients’ poor mental and physical health [59]. Furthermore, recent evidence suggests a shared genetic contribution to loneliness and BPD [60]. Thus, existing interventions for BPD should actively address social connection in treatment and consider it an important marker of recovery [59]. The present study will precisely explore if repeated tDCS sessions over the rVLPFC can mitigate BPD patients’ feelings of social disconnection in daily interactions.

Finally, we will explore whether the reduction in the biased affective interpretation of social events as being rejections, promoted by tDCS, will also favor a decrease in the typical maladaptive behavioral responses to perceived social exclusion exhibited by BPD patients, such as aggressive reactions. These reactions may result in a vicious cycle that might often promote actual social rejection [27,61,62,63], further worsening BPD patients’ emotional dysregulation and interpersonal dysfunction, which account for BPD patients’ higher functional disability compared to other mental and physical disorders [64].

BPD represents a significant public health concern, with a high prevalence in the general population, ranging from 0.7% to 2.7%, and important societal costs [1]. While psychotherapy is the standard of care for treating BPD, it has a limited effect on social adjustment [65] and up to 50% of BPD patients do not sufficiently respond to it [66]. Furthermore, no evidence is available consistently showing that pharmacotherapy is effective in decreasing BPD core symptoms [1,67]. Thus, the finding that tDCS decreases the cascade of interpersonal difficulties associated with BPD during real social encounters may bridge a significant gap in the treatment provision for this severely functionally impaired patient population.

Finally, despite considerable research, the neurobiological underpinnings of BPD have yet to be fully elucidated [1]. For instance, existing research about BPD patients’ neural responses to various degrees of social inclusion yield mixed results in terms of the brain regions involved, possibly due to the diversity of the paradigms that were used [7,9,68,69,70]. In this regard, the results of this study could also inform future neuroimaging and brain modulation studies aimed at elucidating the neural networks involved in the responses to social exclusion and inclusion in BPD. Recent models on the neural correlates of emotion regulation suggest that the ventral and medial portion of the prefrontal cortex might play a more crucial role in emotion reactivity, whereas the dorsolateral portion plays a role in effortful emotion regulation [71,72]. The results might prompt the selection of rVLPFC as an alternative brain target for neurostimulation studies to tap emotion reactivity in place of the most used DLPFC target.

This proposal builds on a well-established line of laboratory research, introducing an innovative focus on longitudinal effects and translational potential. By targeting specific symptoms with non-invasive brain modulation interventions, it aligns with the current surge in precision medicine for mental disorders.

## References

[1] Leichsenring F., Fonagy P., Heim N., Kernberg O.F., Leweke F., Luyten P., Salzer S., Spitzer C., Steinert C. (2024). Borderline Personality Disorder: A Comprehensive Review of Diagnosis and Clinical Presentation, Etiology, Treatment, and Current Controversies. World Psychiatry.

[2] Gunderson J.G. (2007). Disturbed Relationships as a Phenotype for Borderline Personality Disorder. Am. J. Psychiatry.

[3] Gunderson J.G., Herpertz S.C., Skodol A.E., Torgersen S., Zanarini M.C. (2018). Borderline Personality Disorder. Nat. Rev. Dis. Primers.

[4] Hanegraaf L., van Baal S., Hohwy J., Verdejo-Garcia A. (2021). A Systematic Review and Meta-Analysis of ‘Systems for Social Processes’ in Borderline Personality and Substance Use Disorders. Neurosci. Biobehav. Rev..

[5] Cavicchioli M., Maffei C. (2020). Rejection Sensitivity in Borderline Personality Disorder and the Cognitive–Affective Personality System: A Meta-Analytic Review. Pers. Disord..

[6] Williams K.S., Yeager D.S., Cheung C.K.T., Choi W. (2012). Cyberball (Version 5.0) [Software]. https://www.millisecond.com/download/library/cyberball.

[7] Gutz L., Renneberg B., Roepke S., Niedeggen M. (2015). Neural Processing of Social Participation in Borderline Personality Disorder and Social Anxiety Disorder. J. Abnorm. Psychol..

[8] Jobst A., Albert A., Bauriedl-Schmidt C., Mauer M.C., Renneberg B., Buchheim A., Sabass L., Falkai P., Zill P., Padberg F. (2014). Social Exclusion Leads to Divergent Changes of Oxytocin Levels in Borderline Patients and Healthy Subjects. Psychother. Psychosom..

[9] Domsalla M., Koppe G., Niedtfeld I., Vollstädt-Klein S., Schmahl C., Bohus M., Lis S. (2014). Cerebral Processing of Social Rejection in Patients with Borderline Personality Disorder. Soc. Cogn. Affect. Neurosci..

[10] De Panfilis C., Riva P., Preti E., Cabrino C., Marchesi C. (2015). When Social Inclusion Is Not Enough: Implicit Expectations of Extreme Inclusion in Borderline Personality Disorder. Pers. Disord..

[11] Gerra M.L., Ardizzi M., Martorana S., Leoni V., Riva P., Preti E., Marino B.F.M., Ossola P., Marchesi C., Gallese V. (2021). Autonomic Vulnerability to Biased Perception of Social Inclusion in Borderline Personality Disorder. Borderline Personal. Disord. Emot. Dysregul..

[12] Weinbrecht A., Niedeggen M., Roepke S., Renneberg B. (2018). Feeling Excluded No Matter What? Bias in the Processing of Social Participation in Borderline Personality Disorder. NeuroImage Clin..

[13] Kulakova E., Graumann L., Cho A.B., Deuter C.E., Wolf O.T., Roepke S., Otte C., Wingenfeld K. (2024). Evidence of Deviant Parasympathetic Response to Social Exclusion in Women with Borderline Personality Disorder. Eur. Arch. Psychiatry Clin. Neurosci..

[14] Lisco A., Gallucci A., Fabietti C., Fornaroli A., Marchesi C., Preti E., Riva P., De Panfilis C., Romero Lauro L.J. (2025). Reduction of Rejection-Related Emotions by Transcranial Direct Current Stimulation over Right Ventrolateral Prefrontal Cortex in Borderline Personality Disorder: A Double-Blind Randomized Pilot Study. Psychiatry Clin. Neurosci..

[15] Eisenberger N.I., Lieberman M.D., Williams K.D. (2003). Does Rejection Hurt? An fMRI Study of Social Exclusion. Science.

[16] Berkman E.T., Lieberman M.D. (2009). Using Neuroscience to Broaden Emotion Regulation: Theoretical and Methodological Considerations. Soc. Personal. Psychol. Compass.

[17] Onoda K., Okamoto Y., Nakashima K., Nittono H., Yoshimura S., Yamawaki S., Yamaguchi S., Ura M. (2010). Does Low Self-Esteem Enhance Social Pain? The Relationship between Trait Self-Esteem and Anterior Cingulate Cortex Activation Induced by Ostracism. Soc. Cogn. Affect. Neurosci..

[18] He Z., Lin Y., Xia L., Liu Z., Zhang D., Elliott R. (2018). Critical Role of the Right VLPFC in Emotional Regulation of Social Exclusion: A tDCS Study. Soc. Cogn. Affect. Neurosci..

[19] Riva P., Romero Lauro L.J., DeWall C.N., Bushman B.J. (2012). Buffer the Pain Away: Stimulating the Right Ventrolateral Prefrontal Cortex Reduces Pain Following Social Exclusion. Psychol. Sci..

[20] Riva P., Romero Lauro L.J., DeWall C.N., Chester D.S., Bushman B.J. (2015). Reducing Aggressive Responses to Social Exclusion Using Transcranial Direct Current Stimulation. Soc. Cogn. Affect. Neurosci..

[21] Riva P., Romero Lauro L.J., Vergallito A., DeWall C.N., Bushman B.J. (2015). Electrified Emotions: Modulatory Effects of Transcranial Direct Stimulation on Negative Emotional Reactions to Social Exclusion. Soc. Neurosci..

[22] Stoffers-Winterling J.M., Storebø O.J., Kongerslev M.T., Faltinsen E., Todorovac A., Sedoc Jørgensen M., Sales C.P., Edemann Callesen H., Pereira Ribeiro J., Völlm B.A. (2022). Psychotherapies for Borderline Personality Disorder: A Focused Systematic Review and Meta-Analysis. Br. J. Psychiatry.

[23] Nitsche M.A., Liebetanz D., Antal A., Lang N., Tergau F., Paulus W. (2003). Modulation of Cortical Excitability by Weak Direct Current Stimulation—Technical, Safety and Functional Aspects. Suppl. Clin. Neurophysiol..

[24] Nitsche M.A., Cohen L.G., Wassermann E.M., Priori A., Lang N., Antal A., Paulus W., Hummel F., Boggio P.S., Fregni F. (2008). Transcranial Direct Current Stimulation: State of the Art 2008. Brain Stimul..

[25] Benster L.L., Weissman C.R., Stolz L.A., Daskalakis Z.J., Appelbaum L.G. (2023). Pre-Clinical Indications of Brain Stimulation Treatments for Non-Affective Psychiatric Disorders: A Status Update. Transl. Psychiatry.

[26] Lisoni J., Barlati S., Deste G., Ceraso A., Nibbio G., Baldacci G., Vita A. (2022). Efficacy and Tolerability of Brain Stimulation Interventions in Borderline Personality Disorder: State of the Art and Future Perspectives—A Systematic Review. Prog. Neuropsychopharmacol. Biol. Psychiatry.

[27] Preti E., Richetin J., Poggi A., Fertuck E. (2023). A Model of Trust Processes in Borderline Personality Disorder: A Systematic Review. Curr. Psychiatry Rep..

[28] Cain N.M., Meehan K.B., Roche M.J., Sowislo J., Lenzenweger M.F., Clarkin J.F. (2025). From Bench to Bedside: Examining the Interpersonal and Affective Context of Borderline Personality Disorder as It Unfolds over Time in Psychotherapy. OSF Preprints. https://osf.io/fd84s/?view_only=7b7e96d27c1b4aff96f09e0945a3b955.

[29] Davanzo A.D., Huart D., Seker S., Moessner M., Zimmermann R., Schmeck K., Behn A. (2023). Study Features and Response Compliance in Ecological Momentary Assessment Research in Borderline Personality Disorder: Systematic Review and Meta-analysis. J. Med. Internet Res..

[30] Vachon H., Viechtbauer W., Rintala A., Myin-Germeys I. (2019). Compliance and retention with the experience sampling method over the continuum of severe mental disorders: Meta-analysis and recommendations. J. Med. Internet Res..

[31] Faul F., Erdfelder E., Lang A.-G., Buchner A. (2007). G*Power 3: A Flexible Statistical Power Analysis Program for the Social, Behavioral, and Biomedical Sciences. Behav. Res. Methods.

[32] Bolger N., Stadler G., Laurenceau J.-P., Mehl M.R., Conner T.S. (2011). Power Analysis for Intensive Longitudinal Studies. Handbook of Research Methods for Studying Daily Life.

[33] Maas C.J.M., Hox J.J. (2005). Sufficient Sample Sizes for Multilevel Modeling. Methodology.

[34] Kleiman E., Power Curves for Multi-Level Studies (2021). Kleiman Lab. https://kleimanlab.org/resources/power-curves/.

[35] American Psychiatric Association (2013). Diagnostic and Statistical Manual of Mental Disorders (DSM-5^®^).

[36] First M.B., Williams J.B., Benjamin L.S., Spitzer R.L. (2016). SCID-5-PD: Structured Clinical Interview for DSM-5^®^ Personality Disorders.

[37] First M.B., Williams J.B.W., Karg R.S., Spitzer R.L. (2016). Structured Clinical Interview for DSM-5.

[38] Gratz K.L., Roemer L. (2004). Multidimensional Assessment of Emotion Regulation and Dysregulation: Development, Factor Structure, and Initial Validation of the Difficulties in Emotion Regulation Scale. J. Psychopathol. Behav. Assess..

[39] Berenson K.R., Gyurak A., Ayduk Ö., Downey G., Garner M.J., Mogg K., Bradley B.P., Pine D.S. (2009). Rejection Sensitivity and Disruption of Attention by Social Threat Cues. J. Res. Pers..

[40] London B., Downey G., Bonica C., Paltin I. (2007). Social Causes and Consequences of Rejection Sensitivity. J. Res. Adolesc..

[41] Zimmer-Gembeck M.J., Nesdale D. (2013). Anxious and Angry Rejection Sensitivity, Social Withdrawal, and Retribution in High and Low Ambiguous Situations. J. Pers..

[42] Zimmer-Gembeck M.J., Nesdale D., Webb H.J., Khatibi M., Downey G. (2016). A Longitudinal Rejection Sensitivity Model of Depression and Aggression: Unique Roles of Anxiety, Anger, Blame, Withdrawal and Retribution. J. Abnorm. Child Psychol..

[43] Fonagy P., Luyten P., Moulton-Perkins A., Lee Y.W., Warren F., Howard S., Ghinai R., Fearon P., Lowyck B. (2016). Development and Validation of a Self-Report Measure of Mentalizing: The Reflective Functioning Questionnaire. PLoS ONE.

[44] Morandotti N., Brondino N., Merelli A., Boldrini A., De Vidovich G.Z., Ricciardo S., Abbiati V., Ambrosi P., Caverzasi E., Fonagy P. (2018). The Italian Version of the Reflective Functioning Questionnaire: Validity Data for Adults and Its Association with Severity of Borderline Personality Disorder. PLoS ONE.

[45] Morey L.C. (1991). Personality Assessment Inventory (PAI): Professional Manual.

[46] Buckley K.E., Winkel R.E., Leary M.R. (2004). Reactions to Acceptance and Rejection: Effects of Level and Sequence of Relational Evaluation. J. Exp. Soc. Psychol..

[47] Aron A., Aron E.N., Smollan D. (1992). Inclusion of Other in the Self Scale and the Structure of Interpersonal Closeness. J. Pers. Soc. Psychol..

[48] Allen A.B., Leary M.R. (2010). Reactions to Others’ Selfish Actions in the Absence of Tangible Consequences. Basic Appl. Soc. Psychol..

[49] Gandiga P.C., Hummel F.C., Cohen L.G. (2006). Transcranial DC Stimulation (tDCS): A Tool for Double-Blind Sham-Controlled Clinical Studies in Brain Stimulation. Clin. Neurophysiol..

[50] Lefaucheur J.-P., Antal A., Ayache S.S., Benninger D.H., Brunelin J., Cogiamanian F., Cotelli M., De Ridder D., Ferrucci R., Langguth B. (2017). Evidence-Based Guidelines on the Therapeutic Use of Transcranial Direct Current Stimulation (tDCS). Clin. Neurophysiol..

[51] Fregni F., El-Hagrassy M.M., Pacheco-Barrios K., Carvalho S., Leite J., Simis M., Brunelin J., Nakamura-Palacios E.M., Marangolo P., Venkatasubramanian G. (2021). Evidence-Based Guidelines and Secondary Meta-Analysis for the Use of Transcranial Direct Current Stimulation in Neurological and Psychiatric Disorders. Int. J. Neuropsychopharmacol..

[52] Riva P., Gabbiadini A., Romero Lauro L.J., Andrighetto L., Volpato C., Bushman B.J. (2017). Neuromodulation Can Reduce Aggressive Behavior Elicited by Violent Video Games. Cogn. Affect. Behav. Neurosci..

[53] Jung Y.J., Kim J.H., Im C.H. (2013). COMETS: A MATLAB Toolbox for Simulating Local Electric Fields Generated by Transcranial Direct Current Stimulation (tDCS). Biomed. Eng. Lett..

[54] Vergallito A., Riva P., Pisoni A., Romero Lauro L.J. (2018). Modulation of Negative Emotions through Anodal tDCS over the Right Ventrolateral Prefrontal Cortex. Neuropsychologia.

[55] Bikson M., Name A., Rahman A. (2013). Origins of Specificity during tDCS: Anatomical, Activity-Selective, and Input-Bias Mechanisms. Front. Hum. Neurosci..

[56] Siebner H.R., Hartwigsen G., Kassuba T., Rothwell J.C. (2009). How Does Transcranial Magnetic Stimulation Modify Neuronal Activity in the Brain? Implications for Studies of Cognition. Cortex.

[57] Antal A., Alekseichuk I., Bikson M., Brockmöller J., Brunoni A.R., Chen R., Cohen L.G., Dowthwaite G., Ellrich J., Flöel A. (2017). Low Intensity Transcranial Electric Stimulation: Safety, Ethical, Legal Regulatory and Application Guidelines. Clin. Neurophysiol..

[58] Fertonani A., Ferrari C., Miniussi C. (2015). What do you feel if I apply transcranial electric stimulation? Safety, sensations and secondary induced effects. Clini. Neurophysiol..

[59] Mermin S.A., Steigerwald G., Choi-Kain L.W. (2025). Borderline Personality Disorder and Loneliness: Broadening the Scope of Treatment for Social Rehabilitation. Harv. Rev. Psychiatry.

[60] Schulze A., Streit F., Zillich L., Awasthi S., Hall A.S.M., Jungkunz M., Kleindienst N., Frank J., Schwarze C.E., Dahmen N. (2023). Evidence for a Shared Genetic Contribution to Loneliness and Borderline Personality Disorder. Transl. Psychiatry.

[61] Schmahl C., Herpertz S.C., Bertsch K., Ende G., Flor H., Kirsch P., Lis S., Meyer-Lindenberg A., Rietschel M., Schneider M. (2014). Mechanisms of Disturbed Emotion Processing and Social Interaction in Borderline Personality Disorder: State of Knowledge and Research Agenda of the German Clinical Research Unit. Borderline Personal. Disord. Emot. Dysregul..

[62] Fertuck E.A., Preti E. (2023). Interpersonal Trust and Borderline Personality Disorder: Insights from Clinical Practice and Research: Introduction. J. Pers. Disord..

[63] Gutz L., Roepke S., Renneberg B. (2016). Cognitive and Affective Processing of Social Exclusion in Borderline Personality Disorder and Social Anxiety Disorder. Behav. Res. Ther..

[64] Thadani B., Pérez-García A.M., Bermúdez J. (2022). Functional Impairment in Borderline Personality Disorder: The Mediating Role of Perceived Social Support. Front. Psychol..

[65] Storebø O.J., Stoffers-Winterling J.M., Völlm B.A., Kongerslev M.T., Mattivi J.T., Jørgensen M.S., Faltinsen E., Todorovac A., Sales C.P., Callesen H.E. (2020). Psychological Therapies for People with Borderline Personality Disorder. Cochrane Database Syst. Rev..

[66] Woodbridge J., Townsend M., Reis S., Singh S., Grenyer B.F. (2022). Non-Response to Psychotherapy for Borderline Personality Disorder: A Systematic Review. Aust. N. Z. J. Psychiatry.

[67] Stoffers-Winterling J.M., Storebø O.J., Pereira Ribeiro J., Kongerslev M.T., Völlm B.A., Mattivi J.T., Faltinsen E., Todorovac A., Jørgensen M.S., Callesen H.E. (2022). Pharmacological Interventions for People with Borderline Personality Disorder. Cochrane Database Syst. Rev..

[68] Látalová A., Radimecká M., Lamoš M., Jáni M., Damborská A., Theiner P., Bartečková E., Bartys P., Vlčková H., Školiaková K. (2023). Neural Correlates of Social Exclusion and Overinclusion in Patients with Borderline Personality Disorder: An fMRI Study. Borderline Personal. Disord. Emot. Dysregul..

[69] Fertuck E.A., Stanley B., Kleshchova O., Mann J.J., Hirsch J., Ochsner K., Pilkonis P., Erbe J., Grinband J. (2023). Rejection Distress Suppresses Medial Prefrontal Cortex in Borderline Personality Disorder. Biol. Psychiatry Cogn. Neurosci. Neuroimaging.

[70] Ruocco A.C., Medaglia J.D., Tinker J.R., Ayaz H., Forman E.M., Newman C.F., Williams J.M., Hillary F.G., Platek S.M., Onaral B. (2010). Medial Prefrontal Cortex Hyperactivation during Social Exclusion in Borderline Personality Disorder. Psychiatry Res..

[71] Phillips M.L., Drevets W.C., Rauch S.L., Lane R. (2003). Neurobiology of Emotion Perception I: The Neural Basis of Normal Emotion Perception. Biol. Psychiatry.

[72] Phillips M.L., Ladouceur C.D., Drevets W.C. (2008). A Neural Model of Voluntary and Automatic Emotion Regulation: Implications for Understanding the Pathophysiology and Neurodevelopment of Bipolar Disorder. Mol. Psychiatry.

